# Early Interventions for Preventing Self-Harm Recurrence: A Systematic Review and Meta-Analysis

**DOI:** 10.1192/j.eurpsy.2025.336

**Published:** 2025-08-26

**Authors:** G. P. Roncete, L. C. Farhat, L. Beiram, K. L. Ramirez, M. H. Bloch, R. F. Damiano, E. C. Miguel

**Affiliations:** 1 Faculdade de Medicina da Universidade de São Paulo; 2 Departamento e Instituto de Psiquiatria, Hospital das Clínicas da Faculdade de Medicina da Universidade de São Paulo, São Paulo, Brazil; 3 Yale Child Study Center, Yale University School of Medicine, New Haven, United States

## Abstract

**Introduction:**

Deliberate self-harm (DSH) is a strong predictor of future suicide attempts (SAs), highlighting the need to evaluate interventions that reduce recurrence risk.

**Objectives:**

This systematic review and meta-analysis aimed to evaluate pharmacological and non-pharmacological interventions for preventing repeated DSH in individuals recently engaged in DSH. We also examined how participants’ characteristics and trial inclusion criteria impacted interventions’ effectiveness.

**Methods:**

Randomized controlled trials (RCTs) with participants engaged in DSH within one month before enrollment were included. Studies with participants showing only suicidal ideation or older DSH were excluded. We searched five databases (PubMed, Embase, PsycINFO, WHO ICTRP, and ClinicalTrials.gov) with no language restrictions through September 2023. Two reviewers independently selected studies and resolved discrepancies through discussion. Outcomes were assessed at three time points: T1 (0–6 months), T2 (6–12 months), and T3 (>12 months). The primary outcome was repeat DSH; secondary outcomes included suicidal ideation, suicide deaths, all-cause mortality, anxiety, depression, hopelessness, and quality of life. Interventions were categorized by type and delivery format (individual or group, remote or in-person).

**Results:**

The PRISMA flowchart is shown in Image 1. Sixty-one trials involving 125 comparisons and 21,147 participants were included in this review. Average age was 31.5 years, with most being female (66.58%), unmarried (60.82%), and white (65.49%). Participants’ history of self-harm was noted in 48 studies, with at least 56% having engaged in another act before inclusion. In 42 studies, suicidal intent was either not investigated or not required. The most common types of interventions were Treatment As Usual (TAU) (46 arms), Psychotherapy (36), and Active Contact and Follow-up (25). Interventions were primarily applied individually and in person (84), with Cognitive Behavioral Therapy (16) being the most frequent psychotherapy framework. Psychotherapies were associated with a reduced risk of new self-harm acts within the first six months (T1) compared to TAU (k = 12; N = 2110; OR = 0.69; 95% CI 0.48–0.99). Active contact and follow-up interventions outperformed TAU in the longer-term follow-up (T3) (k = 4; N = 3724; OR = 0.83; 95% CI 0.70–0.99).

**Image 1:**

**Figure:**
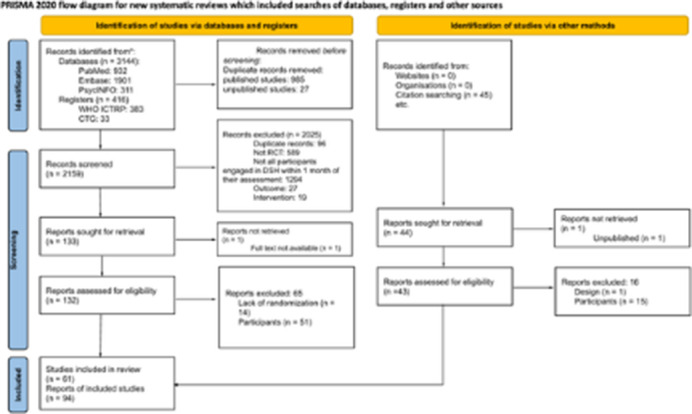

**Conclusions:**

The superiority of psychotherapies at six months was not sustained at twelve months. However, active contact and follow-up interventions may offer benefits in reducing the risk of self-harm recurrence after one year.

**Disclosure of Interest:**

None Declared

